# Violence Against Women in Cambodia: Towards a Culturally Responsive Theory of Change

**DOI:** 10.1007/s11013-017-9564-5

**Published:** 2018-01-17

**Authors:** Maurice Eisenbruch

**Affiliations:** 1 Department of Psychiatry, School of Clinical Sciences at Monash Health, Monash University, Melbourne, Australia; 2 Royal University of Phnom Penh, Phnom Penh, Cambodia

**Keywords:** Gender-based violence, Violence against women, Cambodia, Theory of Change, Ethnography, Buddhism, Violence

## Abstract

Almost one in four women in Cambodia is a victim of physical, emotional or sexual violence. This article brings together two seldom connected fields: Theory of Change (ToC) and cultural responsiveness in international development. It applies these approaches to a priority in global health, which is to prevent violence against women (VAW) and, drawing on my research on the epigenesis of VAW in Cambodia, develops an argument on the need for interventions to work with tradition and culture rather than only highlight it in problematic terms. The research draws on an ethnographic study carried out in Cambodia with 102 perpetrators and survivors of emotional, physical and sexual VAW and 228 key informants from the Buddhist and healing sectors. The eight ‘cultural attractors’ identified in the author’s prior research highlight the cultural barriers to acceptance of the current Theory of Change. ToC for VAW prevention in Cambodia seems to assume that local culture promotes VAW and that men and women must be educated to eradicate the traditional gender norms. There is a need for interventions to work with tradition and culture rather than only highlight it in problematic terms. The cultural epigenesis of VAW in Cambodia is an insight which can be used to build culturally responsive interventions and strengthen the primary prevention of VAW.

## Background

This article brings together two seldom connected fields: Theory of Change (ToC) and cultural responsiveness in international development. It applies these approaches to a priority in global health, which is to prevent violence against women (VAW) and, drawing on my research on the epigenesis of VAW in Cambodia, develops an argument on the need for interventions to work with tradition and culture rather than only highlight it in problematic terms.

ToC, the first seat at the table, is about understanding the best sequence of events that will lead to a desired outcome (Vogel 2012), such as the prevention of VAW, and how that change will happen. Vogel’s review report for DFID captures the elements. ToC approaches have moved into the mainstream in international development and a key driver for ToC should be country-owned development, with collaboration with local actors.

Turning to VAW, global efforts to prevent it have been fuelled by a contemporary ToC, developed in the west and applied globally. What is appealing about ToC is that, with an open learning approach, it is supposed to make these assumptions explicit, and to activate and support critical thinking throughout the program so that dynamic changes can be made in response to changes in contexts (Vogel 2012).

Seldom, however, do agencies pause to consider whether the assumptions are appropriate in culturally diverse settings. This is probably because what people in a group believe to be true about the genesis of VAW can be summarised as ‘culture A engages in harmful traditional practices’ or ‘culture B champions male hegemony in which men can abuse women’ or ‘culture C teaches women that they have next-to-no human rights’ and ‘in a rapidly changing social-economic structure, men in culture D cannot cope with the increasing independence of women and therefore seek to control them through violence’. Armed with these sorts of assumptions, and with little debate, policy makers and program developers design initiatives to tackle the problem.

The second seat at the table is cultural responsiveness in international development and evaluation. Chouinard is scathing in her criticism of international development evaluation for generally omitting culture ‘despite the recognition that evaluation is an intensely cultural practice’ (Chouinard 2016:237). Chouinard offers an invaluable conceptual framework for locating culture in international development, from which I select a few questions pertinent to this article. The epistemological dimension of cultural practice includes western versus local approaches to knowledge construction, with key questions such as which forms of knowledge are privileged, dominant or excluded and whose voices and perspectives frame the analysis or are excluded. The ecological dimension includes the broad social and economic influences, with key questions such as to what extent the history, culture and background of the local community informs the design, process and consequences of the evaluation. The methodological dimension includes the range of philosophical approaches, levels of exclusion and voice, and the multicultural validity of the data, with key questions such as whether validity is defined in culturally appropriate ways.

My argument is that, for ToC to be culturally responsive, there has to be some grasp of the ‘internal architecture’ of VAW. With that point of departure in mind, I am taking as a benchmark my previous work in which I describe the cultural epigenesis of VAW in Cambodia. I depicted eight ‘cultural attractors’, starting with blighted endowment, or ‘bad building’ (*sɑmnaaŋ mɨn lʔɑɑ*), determined by deeds in a previous life (*kam*). Then I discussed the perceived role of vicious character in early life (*kmeeŋ kaac* or *doṣa*-*carita*) that might lead to a person becoming an abuser, and particular birthmarks on boys which are thought to be portents. Next, I discussed the powerful local concept of *krʊəh*, or mishap, especially when a female’s horoscope predicted a zodiac house on the descent (*riesəy*), as explaining vulnerability to violence and its timing. Given the importance of marital harmony, I then considered astrological incompatibility (*kuu kam*) as a risk factor and what people seem to feel can be done about it. I then presented a particularly Buddhist perception that GBV is fuelled by lust, anger and ignorance, the ‘Triple Poison’. The usual suspects of alcohol and poverty were considered in the next cultural attractor, called ‘entering the road to ruin’ (*apāyamuk*). The two final cultural attractors I presented have to do with the mental state of a perpetrator, depicted here as confusion and loss of judgement (*mohā*), followed by moral blindness (*mo baŋ*). In an epigenetic formulation, I showed how the ‘cultural attractors’ make up the landscape of GBV.

VAW around the world is culturally constructed. Examples of such studies include the Arab world (Standish 2014), Bangladesh (Fattah and Camellia 2017; Chowdhury 2014), Bosnia (Muftić and Bouffard 2008), China (Tang, Wong, and Cheung 2002), India and China (Taylor, Xia, and Do 2017), Myanmar (Norsworthy and Khuankaew 2004), Nepal (Puri, Shah, and Tamang 2010), Nigeria (Oladepo, Yusuf, and Arulogun 2011), Pakistan (Sadiq 2017), Rwanda (Zraly and Nyirazinyoye 2010), Senegal (Bop 2010), Thailand (Ezard 2014; Han and Resurreccion 2008), Turkey (Ozcakir et al. 2008) and Vietnam (Krause et al. 2015). Despite the undeniable role of culture, a role that is universally acknowledged, seldom does it find an easy home in the discourse on GBV. Heise (1998), in her seminal work on understanding the origins of gender-based violence, proposes an ecological framework that gives equal voice to disciplines such as psychology and sociology or the feminist emphasis on gender inequalities and power differentials. Heise’s framework inspires the hope that a culturally responsive approach to GBV can interweave with the individual ontogenetic, microsystem situational, exosytem and macrosystem factors. Years later, Fulu and Miedema (2015) updated Heise’s ecological model by incorporating globalized ideologies, economic development and integration, religious fundamentalisms, and global cultural exchange as components of a larger globalization process, to explore the relationships between global processes and experiences of VAW. My deduction is that working with local cultural brokers and religious leaders is essential to advance culturally informed strategies to prevent GBV. Petersen has recently warned that, in post-apartheid South Africa, ‘feminist theologians and practitioners note that although the rights discourse is an essential democratic value …it is not in itself an effective intervention strategy in contexts where religion (embedded in culture) is a social determinant of hierarchical gender power-relations’ (Petersen 2017:1).

O’Brien and Macey, in their review of 15 articles to examine GBV interventions, note that, unfortunately, most interventions have been developed for dominant culture populations and that there are hardly any culturally specific interventions for survivors of minority cultural or ethnic groups (O’Brien and Macy 2016). The exceptions they reported were studies based in Sierra Leone that explored the use of traditional healers and the need for intervention to include religious references. What is more, they warn, interventions developed for survivors in the dominant culture are increasingly used in nondominant cultures. They argue that local informants are essential not only because they provide inside knowledge about local traditions regarding GBV, but also because they can identify acceptable intervention formats and approaches.

### Cambodia

I want to suggest how local cultural insights into VAW might be used to work with tradition and culture rather than only highlight it in problematic terms, and thereby achieve a more culturally responsive approach to Theory of Change, an outcomes-based approach increasingly used in international development, reflecting a need to re-emphasise the deeper analysis of ‘how to get to our objectives’, and which has come to shape national action planning (Vogel 2012).

#### Violence, Culture and Buddhism

This atmosphere of violence might seem surprising in a country where Buddhism is the national religion, and where, from time to time, Cambodian Buddhism has connected with radical religious narratives for peace. Monks and female devotees have been involved in ‘engaged Buddhism’—the contemporary movement of nonviolent social and political activism found throughout the Buddhist world (King 2005)—applying the insights from *dhamma* teachings to circumstances of social injustice and structural violence (Rothberg 1997). Paradoxically, the Khmer Rouge had its origins in the Buddhist nationalism of the 1940s, and Cambodia was among the countries where, in the post-colonialist phase, there were efforts to merge Buddhism with socialist ideals (Ladwig and Shields 2014).

#### Violence Against Women in Cambodia

The levels of VAW in Cambodia are alarming (Fulu et al. 2015) and often not reported (UN Women 2014). The regional UN Multi-Country Study on Men and Violence survey (Fulu et al. 2013b) found that 32.8 per cent of men in Cambodia reported perpetrating physical and/or sexual violence against an intimate partner in their lifetime, and one in five men reported raping a woman or girl, one of the highest recorded rates in the Asia–Pacific region (Fulu et al. 2013a:9). Papua New Guinea-Bougainville, Indonesia-Papua and Cambodia have the highest rates of gang rape (*bauk* in Khmer) in the region. Gang rape in Cambodia is reported to be linked with aspects of the culture related to sexual entitlement (Wilkinson, Bearup, and Soprach 2005) and prompted by viewing gang rape pornography (Farley et al. 2012). Fulu and Miedema (2015) point out that the situation has been made both worse and better by the effects of globalisation: economic development has meant that women have flocked to the cities in search of work, a situation that prompts more GBV; at the same time, the heightened awareness and activism arising from the global women’s rights discourse has brought GBV to the fore as a social issue of the highest importance.

This bleak picture has spurred action by the government of Cambodia, which has signalled its commitment to responding to GBV. Among several UN instruments, in 1999, it ratified the Convention of the Elimination of All Forms of Violence Against Women (CEDAW) and in 2010 signed its Optional Protocol (Fulu et al. 2015). Cambodia has a national legal and policy framework to protect women’s rights, including the Law on the Prevention of Domestic Violence and Protection of Victims of 2005, the 2007 Criminal Procedure Code, the Law on Suppression of Trafficking in Humans and Sexual Exploitation of 2008 and the National Action Plan to Prevent Violence Against Women 2013–2017 (Ministry of Women’s Affairs 2014). The EVAW program of the Australian government (Australian Aid 2016) funded the first National Survey on Women’s Health and Life Experiences in Cambodia, 2014. The outcome areas were services (counselling and responses to mental health problems and support and referral for sexual harassment and domestic violence), prevention (training and community awareness through events and forums to influence knowledge, attitudes and behaviours in responding to and understanding the impact of VAW) and justice (legal intervention combining capacity building activities for local authorities to better understand their legal obligations in responding to VAW and the provision of legal services for victim of GBV). The Ministry of Women’s Affairs (MoWA) developed and implemented the second National Action Plan on Violence Against Women 2014–2018. The Referral Guidelines for Women and Girl Survivors of Gender-Based Violence promote access to services through a system of case registration, assessment and referral based on the individual needs and agreement of the survivor, recognizing that survivors of GBV have multiple needs that cannot be met by any one service provider.

Collaboration and coordination between key service providers and agencies has resulted in the development and implementation of a number of practice guidelines to support service providers in delivering services to the victims of violence and these guidelines complement the existing legal framework in Cambodia. Data Management System is a common system of reporting on service provided to better understand service requirements, service gaps and blockages. The Minimum Service Standard for Basic Counselling of Women Survivors of Gender-Based Violence provides guidance to all service providers to ensure a common set of principles to facilitate privacy, confidentiality and respect for the rights of the survivor to information and to make decisions about their future. The Clinical Handbook of Health Care for Women Subjected to Intimate Partner and Sexual Violence provides guidance for health care providers in health centres and referral hospitals to provide first-line support and clinical management for women victims of sexual violence.

These policy and program advances do not systematically consider culture. Fulu and Miedema report the need for understanding the effect of ‘subjective experiences and local meanings of actors in specific cultural settings’ (Fulu and Miedema 2015:2). Cultural context may influence both the risk of gender-based violence and the cross-cultural differences in the coping styles of people affected by it. There has been a tendency to depict the cultural issues involved in intimate partner violence as outcomes of Cambodia’s hegemonic masculinities. The seminal study by Zimmerman entitled ‘Plates in a basket will rattle’ (Zimmerman 1994) tends to be quoted as clinching the view that Cambodian society automatically accepts the power of men and that violence by men against women is a cultural norm and does not justify intervention (Hill and Ly 2004). Of the more than 35 articles citing Zimmerman, only Brickell has focused on the *actual* meaning of the ‘saying’ adopted by Zimmerman as the title of her article. Brickell calls it ‘an archetypal trope for the inevitability of marital disputes’. It could also refer to gendered issues linked to conjugal failure such as domestic violence (Brickell 2014).

The common wisdom in Cambodia, shared by expatriate experts and the Cambodian leadership, is that cultural acceptance of violence fuels and perpetuates it. Ing Kantha Phavi, Minister for Women’s Affairs, said recently that ‘it is … because of our society and our community’. There is a problem, however, with blaming Cambodian culture and tradition for GBV. Research highlights the ingrained gender-based cultural scripts (Gourley and NGO Committee on the Rights of the Child 2009), which, for men, include the widely assumed ‘hegemonic masculinity’ (Lilja 2011). ‘Hegemonic masculinity’ is normative because it is the most honoured and ‘culturally exalted’ way of being a man, and the assumption is that other men might follow its dictates (Connell and Messerschmidt 2005). Partners for Prevention and Miedema (2011) identified the existence of multiple interpretations and experiences of Cambodian masculinities within the lives of the men interviewed. The many NGOs in Cambodia established for the purpose of ending GBV seek to modify ‘hegemonic masculinity’ and teach women about their human rights, but GBV levels remain very high. All relevant government ministries have signed ‘Core Commitments’ (Royal Government of Cambodia 2014b) to tackle traditional attitudes identified as barriers to behavioural change in men and women (CARE 2014). In this, NGOs seek to persuade men to break with tradition, to ‘rupture’ their ‘repetitions’ of violent masculinities that, according to Lilja and Baaz (2015:2) contribute to acts of violence and at the same time, if targeted, could create possibilities for resisting intimate partner violence.

Literary texts such as the ‘woman’s code’ (*cbap srəy*) reinforce domestic abuse as acceptable (Surtees 2003). Chandler (1984) depicts the *cbap* as gnomic, normative poems popular in pre-colonial Cambodian society and which, unlike chronicles or inscriptions depicted the activity of the entire society rather than that of just the elites, and which helped people confront the harsh realities of everyday family life. Brickell (2011) highlights the different cultural discourses that men and women draw on to explain the persistence of inequality, which she terms a ‘stubborn stain’ on development. The usual suspects—cultural condoning of alcohol abuse, sex outside marriage and challenges to traditional male masculinity—have been well documented (GADC 2010). It has been suggested that men exploit cultural hegemony in various ways, such as by blaming alcohol, or prioritising the harmony of the home over the well-being of women. Brickell and Garrett (2015) used collective storytelling in participatory video workshops to explore the hegemonic norms, and they found that the primary community perceptions of the ‘cause’ was alcohol.

These studies are springboards for further elucidating the cultural construction of VAW—how their causes and consequences are perceived, and how, in this tapestry of violence, the warp of the social-economic-political yarn is interwoven with the weft of cultural meaning. What is at stake is how these cultural insights are used, or bypassed, in developing new theories and approaches to change.

As for Theories of Change, the common wisdom, shared by expatriate experts and the Cambodian leadership, is that cultural acceptance of VAW fuels and perpetuates it. Apart from citing ‘cultural obstacles to violence prevention, because these beliefs hamper the fair treatment of victims’ (Royal Government of Cambodia 2009:4), the Second National Action Plan to Prevent Violence Against Women 2014–2018 (NAPGBV) works to change cultural attitudes through primary prevention and initiatives such as the ‘good men campaign’, which aims to get men to question the norms of masculinity (UNFPA 2015) in order to develop new cultural values and norms that are incompatible with violence. As Partners for Prevention note, ‘gender is essentially a construct of the global North exported to other parts of the world … The question of how this concept can be transferred in a short timeframe and used within a cultural and social context with different circumstances … must be kept at the forefront in any research that deals with gender’ (Manero and Popovici 2010:10).

The Ministry of Health (MoH) has undertaken efforts to translate the guidance into practice, in line with WHO guidelines (2016) and international best practice. WHO (2013) recommends that this training should take cultural competency into account, but the national clinical handbooks and training curricula do not seem to address cultural norms beyond seeking to eradicate them. Normative, feminist and social constructionist Theories of Change do not leave room for the *emic* perspectives of violence. Kent, in her work on gender, security and religion in Cambodia, has warned that ‘what tends to be overlooked from this position, however, is the way in which culture may simultaneously provide avenues for men and women to achieve security in concert with the maintenance of a seemingly androcentric gender order’ (Kent 2011:194).

To provide a comprehensive understanding of the ways in which Cambodians see the causes and effects of VAW in Cambodia and to expose and analyse the cultural forces that underpin and shape its landscape, it is important that everyone working to prevent or mitigate violence in Cambodia be familiar with its local idiom.

The article sets out the epigenesis of VAW. I document the local understanding by perpetrators and survivors and their families, as well as monks and healers, of the causes and effects of intimate partner violence, and include the Buddhist and folk stories they use to describe it. I explore the popular idioms expressed by a range of people to depict violence as they know it and delineate the local symptom patterns of mental and physical distress experienced by survivors and perpetrators.

The article moves through eight ‘cultural attractors’, demonstrating how each works in propelling a person to violence. I discuss the practical implications for counselling, prevention and law enforcement, and discuss the possibilities of applying the cultural epigenesis of GBV for the development of a culturally responsive ‘Theory of Change’.

## Cultural Epigenesis of VAW in Cambodia

The approach and method to the ethnographic research on the cultural epigenesis of VAW in Cambodia is described in the companion article to this one (Eisenbruch 2018). The study involved 102 perpetrators and survivors of emotional, physical and sexual VAW and 228 key informants from the Buddhist and healing sectors.

The results revealed eight ‘cultural attractors’, which for simplicity are set out as eight clusters. The popular cultural perceptions of VAW, in Khmer *ʔɑmpəə həŋsaa ləə strəy*), are deeply embedded for both men and women in Cambodia.

VAW has form and structure. Its epigenesis is pulled into shape by the guy ropes and pegs that connect the cultural attractors and direct the course of the rivulets in the epigenetic landscape, one ‘cultural attractor’ after another. The rivulets could be considered to be the course of individual people’s lives, and they are also larger, the patterns of social and cultural practices. It starts with blighted endowment and continues as seeds of character, emerging as astrological forces. It erupts like poison and develops once the perpetrator enters the road to ruin, a powerful central ‘cultural attractor’. It sets the stage for the act of violence as the perpetrator’s mind becomes confused and seals his fate with the onset of moral blindness and an absence of shame and blame.

Blighted endowment—The popular Cambodian Buddhist view is that those who die a tragic death, especially those whose final moments are violent, will have difficulties making the transition and having a positive rebirth (Holt 2012). Committing violence from beyond the grave is a powerful driver for ‘identifying with the aggressor’ by turning the passive experience of being hurt into the act of hurting others. The millenarian ‘Buddha Predictions’, the *Buddh Damnāy* (de Bernon 1994), forecast endemic violence in Cambodia (Hansen and Ledgerwood 2005). People draw on these predictions as a way of steeling themselves against unbridled violence, including that against women, and their ideas are given expression in the designs known as *yantra*, such as the ‘Three Vast Plains of War, Famine and Disease’ which were drawn by monks to help dispirited survivors (Eisenbruch et al. 2013). Rather than simply befalling individuals, violence can affect large numbers of people or even the nation as a whole.

Character—Character, or *caʔret* is rooted in a person’s predestiny in the previous life (*karma*), coloured by its genetically shaped breeding stock (*puuc*), and further shaped by the way a child has been reared in a particular family milieu (*pʊəŋ*, literally, ‘its pedigree’). Contrary to formal Buddhist doctrine, character is also believed to be shaped by the position of the planets and the stars and the child’s year of birth. A further example of a harbinger of violence thought to be imprinted on the child is the mole. In Buddhist societies, a mole, far from being trivial, was regarded as predictive of the child’s nature in adult life, often a grave portent of character and a marker of violence (Jungwiwattanaporn 2006; Flint 2011).

Astrological misfortune—*Krʊəh* is a concept deeply embedded in the Khmer psyche. It had to do with having been seized by a particular constellation of the nine heavenly bodies and known as *nup krʊəh*. The demon seizes its hold upon the sun or moon—or upon a human ‘seized’ for better or worse by their astrological destiny. Reynolds (2016) describes the sciences of prognostication in Buddhism, such as astrology, and the interpretation of birthmarks, as ‘deployed to help people face up to unpredictability in life’. The results show how people seek to curb astrologically shaped violence in the context of a combination of civil human rights law (*ʔaanaacak*) and Buddhist doctrine (*puttʰeaʔcak*).

Astrological incompatibility—Inauspicious unions, however, are *kuu kam*—prone to misfortune and violence in the course of the marriage. An abuser can project the blame on his wife. The best match, to a bride classified as completely virtuous (*srəy krup leakkʰaʔnaʔ*), would not lead to violence. The alternative would be a woman whose character was not completely virtuous (*srəy kʰaat leakkʰaʔnaʔ*), who would damage the groom’s good fortune (*kʰaat liep*) and make him go down the road to ruin (*apāyamuk*) or, as some said, to defeat and destruction (*paʔraa cey*). As for the character of the husband, he could bolster his character through becoming a monk for a short time, learn Buddhist morals and qualify as a ‘scholar’ (*pandita*). When he left the monkhood, he would be called a completely virtuous man (*proh krup leakkʰaʔnaʔ*) and suitable to be an exemplary husband. The harmonious marriage would be one in which the husband was *proh krup leakkʰaʔnaʔ* and the wife a *srəy krup leakkʰaʔnaʔ*.

Triple poison—It is clear from Buddhist teaching how craving spills into anger and how each is a forerunner of violence. *Mohā*, ignorance and delusion, is the third poison and, in Buddhist teachings, the abuser’s mind becomes deluded through ignorance (*avijjā*), causing perversions that take what is painful (*dukkha*) as pleasurable (Dhammasami et al. 2012). Beating a wife would not trouble the perpetrator in a state of *mohā*. Abusers are oblivious to the reciprocal relationships with their wives as victims.

The road to ruin—Entering the road to ruin (*apāyamuk*) is a powerful peg—possibly the central one—in the epigenesis of violence. Indulgence in ‘alcohol, women, and gambling’—paths likely to be taken by impoverished, unemployed and desperate men and women—invites self-destruction (Kitsripisarn et al. 2013). Buddhism has various taxonomies of *apāyamuk*, a popular one being ‘The Six Bad Habits’: being lazy, gambling, watching bad entertainment, going out late at night, consorting with people of ill-repute and addiction (Radich 2007). Of these, liquor features as the fifth of the Five Precepts to be observed by all Buddhists. Government rhetoric on the perceived causes of domestic violence tends to focus on alcohol and poverty. A raft of national-level surveys has tried to establish the basis of VAW in Cambodia (Banta et al. 2012). Alcohol use is linked to coercive sex and domestic violence. In her work on gendered experiences of drunkenness and violence in Cambodia, Brickell makes plain the complexity in cause-and-effect between alcohol and abuse; she explores the ‘vocabularies of motives’ used to differentiate alcohol use and violence and sees the underlying inequality between men and women, rather than the alcohol itself, as causing gender-based violence (Brickell 2008). An abuser on the road to ruin wrecks the life of his intimate partner.

Moral blindness—*Mo baŋ* means complete darkness, thoughts mixed and muddled, the man not knowing right from wrong, the ‘empty mind’ (*mohā*) becoming shut off (*baŋ*) from awareness (Chuon Nath 1967). An important aspect of moral blindness is the way in which, with time, people develop a shifting pattern of ‘social memory, violence, trauma and morality’ (Kent 2015). Moral blindness leads to feelings of impunity (*nitoandeaʔ pʰiep*), a word that finds its root in ancient usage, meaning ‘the position of no penalty’, literally, ‘no rod’. Cambodians use the Khmer word ‘*touh*’, which is embedded in Indian mythology and Buddhism, and means a ‘pastiche of fault, blame and punishment’ (Headley, Chim, and Soeum 1997). *Hiri ottappa* is a compelling Buddhist explanation for the insouciance of a morally blind person (De Silva 1976). *Hiri* is a healthy sense of shame which deters a person from committing violence. *Ottappa* is the fear of blame or moral dread that deters a person from committing violence through fear of recrimination. While a person is morally blind, he is incapable of noticing *hiri ottappa*.

## Implications

To speak of GBV in Cambodia, it is essential as a starting point to be clear what Cambodians and westerners mean by the term ‘gender’, which the Ministry of Women’s Affairs (2014) defines to be the socially constructed attributes and opportunities associated with being male or female. Here is a challenge, for in Khmer the difference between male and female is expressed by the Khmer *pʰeit*, but there is no word for ‘gender’, and contemporary NGOs have had to adopt the loanword ‘*zenda*’ from the donor language English. As Aveling (2012) showed, ‘gender’ is viewed as a foreign word associated with international NGOSs; the concept remains relatively novel, with some elements being rejected as inapplicable to Khmer society and others hybridized with traditional knowledge.

There are practical implications for counselling, prevention and law enforcement.

### Counselling

- Exploit the perpetrator’s sense that his misfortune arose from having violated a moral ‘code of conduct’. Train counsellors to appreciate that the perpetrator may feel more comfortable in projecting and ‘locating’ the source of his violence outside himself and that he may therefore seek traditional interventions (e.g. substitution rituals) to repair those external sources of violence. Urge him to cease his absence of guilt and remorse and take personal responsibility for his violence.
- Encourage survivor (and perpetrator) to use their own language to give vent to their feelings towards one another (e.g. of suspicion or jealousy) using local idioms as much as possible, so that they can understand their partner’s experience. Exploit the local Cambodian expressions, encouraging survivor (and perpetrator) to use their own metaphorical language of expression of fiery destructive feelings of anger, to help each to understand their partner’s experience.
- Highlight the beliefs of husband and wife, for example, to reinforce their conviction that their adherence to the spiritual codes of conduct of their ancestral spirits must include non-violence, lest those spirits attack them in revenge. Empower husband and wife to combine the best of Western therapy and local practices (*?aakum psɑm ?aayu?*) to resolve mutual tension and anger (*sɑmrɑh samruəl*) by methods they feel might diffuse their anger (*rumsaay kɑmhǝŋ*) rather than bottle it up and vent it on their partner.

### Prevention

- Identify the man’s expressions of his sense of impunity, and his reactions to any perceived threats to his masculinity arising from his wife’s getting work. Use the perpetrator’s choice of verbal expressions/idioms that reflect deep-seated male and female attitudes to masculinity and the roles of husband and wife.
- Be alert to danger signs of impending murderous revenge (e.g. ‘head and tail chopped off’) and intervene to prevent violence. Encourage survivor (and perpetrator) to notice the early warning signs of smouldering repressed anger (e.g. *muə mav*) for example, resentment of changed masculinity in the face of his wife’s getting work, and to take action before it spills out of control into full-blown verbal or physical violence.
- Adapt Western principles of confidentiality to use Buddhist or local notions of ‘shame’ to motivate the perpetrator (and survivor) to reveal violence within the family and accept counselling without loss of face. Research alternatives to instructing and leading people (*nae noam*) and explore whether a perpetrator who feels more immunised against misfortune can become more accepting of education about human rights and his responsibilities to abide by the rule of law.

### Law Enforcement

- Train local authorities and encourage monks to use the perpetrator’s way of thinking about his *apāyamukha* to puncture his sense of impunity, to accept the human rights of the survivor and his liability to law enforcement. Educate people to stop using a ‘cultural defence’ of the perpetrator, because in Buddhism as in international law, they must intervene when a man has criminally attacked his wife.
- Use ideas that may appeal to traditional rural families, for example, the survival of women of virtue who have endured untold violence (e.g. links with popular media such as the *Maranamata* movie). Adapt popular *Vessantara Jataka* legends to deter would-be perpetrators from acting on their jealousy.

## Theory of Change

International development, particularly in the field of GBV, increasingly draws on Theory of Change. It is both a tool and a methodology to map the logical sequence of an initiative from inputs to outputs and a deeper, reflective process and dialogue amongst colleagues and stakeholders that reflects the philosophies and worldviews of change held by people (Vogel 2012). Models of change can and should include the granularity provided by detailed cultural studies such as the one provided by this article. To the best of my knowledge, this article is the first applied research in Southeast Asia that harnesses local culture with a view to primary prevention of GBV and to injecting cultural responsiveness into Theory of Change. Theory of Change prescribes a metamorphosis in ‘values, beliefs, attitudes, behaviours and practices about violence’ (Action Aid and Moosa 2012) as a necessary enabler. The Cambodian government’s Second National Action Plan to Prevent Violence Against Women (Royal Government of Cambodia 2014a) implicitly aims to change cultural attitudes on GBV through initiatives which aim to get men to question norms about masculinity in order to develop new cultural values and norms that are incompatible with violence.

Theory of Change is driving the campaigns to arrest VAW and girls. It is built on norm, feminist and social constructionist theory, which ‘argues that partner violence is in part a function of social norms, as well as structures that grant men the right to control female behaviour and limit women’s power in both public and private life’ (Heise 2011). Brickell (2014) strongly articulates a tension between the international human rights priority on protecting women from abuse and the Cambodian priority on maintaining harmony.

One flaw in contemporary approaches is that where local culture and tradition are seen as hindering change, they are taken as explanations for the gap between formal legislation of international standards and continuing VAW and girls (Brickell, Baureaksmey, and Poch 2014). This is a strange cycle that may keep the work on primary prevention of GBV focused on the wrong target. Prevention of GBV, in any country, has to deal with local tradition and culture, which is dynamic and changing. The challenge is to change the ‘tradition’ of harming women, not to frame it as the barrier to doing so, and to convince the people, one needs to develop a common understanding and create rapport.

We need to be mindful of the dynamics that both propel and inhibit real change. Why is the international community so intent on eradicating the ‘stubborn stains’ of the hegemonic masculinity of men and the passivity of women? Why is there such alacrity among Cambodian gatekeepers to accept the received wisdom embedded in contemporary Theory of Change? And why, in spite of the valiant efforts of government, multilateral agencies and local organisations flowing from the First and Second National Action Plan to Prevent Violence (NAPGBV), do many ordinary men and women remain resistant to change? Not only is this a failure of primary prevention of GBV, it is a waste of money and leads to a demonisation and systematic corrosion of the local fabric of Cambodian culture. Perhaps one reason is that the contemporary Theory of Change is not culturally responsive to the reality in Cambodia. The NAPGBVs requires a Theory of Change built on what makes sense to ordinary men and women, and it may be more practical, as I said before, to make culture and tradition a part of the solution, rather than the core of the problem.

The findings in this article could help make sense of this tension, with implications for a culturally responsive Theory of Change. In mapping the cultural landscape of GBV, the findings tell us that the epigenesis of GBV is pushed and pulled into various shapes by the eight ‘cultural attractors’, one after another. 1) **Karma:** National Action Plans view Cambodian culture as preventing men and women from changing their behaviour because they are doomed from actions in their previous life. *The findings suggest this to be untrue, and competent monks could use karmic theory to show an abuser that he is accountable for his actions and a survivor that she is not to blame.* 2) **Character:** The National Action Plans are silent on the question of the character of abusers or survivors in GBV. *The findings suggest that people regard the character of abusers or survivors as critical to the later development of violence*. Boys and girls may carry physical stigmata from early life, for example, that lead to perpetration of intimate partner violence or even child sexual abuse and incest later in life. 3) **Astrology:** The National Action Plans are silent on the role of astrology. *The findings show that those affected by GBV are mindful of* krʊəh—*the Khmer concept of astrological mishap*—*as a signifier explaining the risk and vulnerability of a girl or a woman to GBV*. Monks can intervene to promote reconciliation. 4) **Marital incompatibility:** The National Action Plans recognise that certain marriages are violence-prone and they encourage divorce when there seems to be no other solution. There is a tension between Theory of Change’s priority to arrest GBV and society’s impetus to maintain ‘harmony’. *The findings on* kuu kam*, the astrologically determined mismatch between husband and wife, shed light on this tension and suggest that monks could intervene to free troubled couples to move on harmoniously together.* Monks could carry out primary prevention by warning that astrology should not be misappropriated as a justification for GBV and that men and women must accept agency for their actions in GBV. 5) **Emotional drivers:** The National Action Plans acknowledge that there are criminal abusers and that they are treated accordingly by the state, but there is no grasp of the cultural drivers at work. *Our findings show the role of Cambodian notions of lust, anger and ignorance, the Buddhist ‘Triple Poison’, an emotional*–*cognitive complex that fuels sexual and physical violence against women and girls.* A related cluster, envy–jealousy, fuels gender-based acid attacks, in particular when a spouse thinks that their partner has been unfaithful, or sorcery, when a man or woman spurned by a hoped-for married lover hires a black magician to cast a spell and wreck the couple’s marriage, often by inducing marital violence. Monks could carry out primary prevention of GBV by promulgating warnings about the Triple Poison and its consequences for GBV. 6) **Social ruin:** The National Action Plans pay much attention to structural factors such as alcohol abuse and changes in hegemonic masculinity as drivers for GBV, but the local cultural context tends to be overlooked. *The findings suggest that alcohol and hegemonic masculinity, and other drivers such as pornography or substance abuse, are better understood through the Cambodian Buddhist view of ‘the road to ruin’ (*apāyamuk). Monks could help to prevent GBV by highlighting the perils of *apāyamuk* and dissuading would-be perpetrators. 7) **The mental state of a perpetrator committing GBV:** The National Action Plans are silent on the mens rea of the offender. *The findings suggest the importance of the perpetrator’s loss of judgement (*mohā*) and failure to grasp the consequences for himself and his family*. 8) **Moral blindness and accountability:** The National Action Plans strive to educate people to be ‘good men’ and ‘women with agency’, but there is no pathway to overcome the inherent cultural resistance to change. *The findings suggest how ‘moral blindness’* (*mo* baŋ) *seems central to feelings of impunity when it comes to GBV*. Recognising that Cambodian ‘culture’ has moved fast in response to neoliberalism, and that the tapestry of spirituality is moving with it, monks and female devotees can use local cultural notions to help bring an end to the culture of impunity and prevent GBV.

In Theory of Change, the Outcomes Pathway is the change logic, the sequence of outcomes needed in a logical relationship, with early outcomes needed before subsequent ones. The eight ‘cultural attractors’ constitute a potential blueprint that could be translated to harness and enhance all components of the Outcomes Pathway—the change logic in which early, intermediate and late outcomes are in place in sequence—and advance a culturally responsive Theory of Change. Taking the second step (barriers) of the Outcomes Pathway, for example, the first four ‘cultural attractors’ identify why, despite many rights-based efforts, people perpetuate the dominant social norms that support violence. The ‘cultural attractors’ explain the barriers that prevent rights-based programs from overcoming the lack of autonomy of those vulnerable to violence. As for the third step (interventions) in the Outcomes Pathway, agencies could harness the knowledge gained by the present study to teach people about human rights, for example, in a way they understand; change social norms without tearing apart the society’s cultural fabric; build institutional capacity by bringing appropriately selected cultural experts such as monks into training; and provide better services by informing prevention, counselling and law enforcement.

Understanding how people view childhood markers of violence (*carita*), or the nature of moral blindness (*mo baŋ*), for example, could help the agencies to understand the barriers to the lack of guilt and remorse and overcoming the feeling of impunity. The first four identify why, despite many rights-based efforts, the people continue to perpetuate the dominant social norms that support violence. The ‘cultural attractors’ also reveal the barriers that rights-based programs encounter in trying to overcome the lack of autonomy of those vulnerable to violence.

The rights-based approaches could be better informed by the styles of the Cambodian Buddhist interventions, such as substitution rituals in which the suffering of a survivor is transferred to an effigy, or in which ritual reparations are made not only to the wronged person but also to their guardian spirits and the cosmic deities in a kind of local cultural nod to the western law of torts. Monks also carry out preventative work by educating communities on non-violence using the simple formulas and precepts of morality in the *dhamma* and expressing the ideas through easily grasped *suttas*, a particularly Cambodian rendition of obligations between people, and between people and the supernatural worlds. We see these teachings at work in real cases of violence in which the monks disabuse the culprit of their sense of entitlement and impunity.

The Buddhist monks and female devotees focused on in this study do not necessarily hold views which take a victim-centred approach. There is pressure from the government for cases involving GBV and domestic violence to be dealt with and ‘disposed of’ at a local level through Alternative Dispute Resolution (ADR) rather than the ‘judicial solution’ (Ministry of Women’s Affairs 2014). According to Article 26 of the DV Law 2005: ‘For the offenses that are the mental/psychological or economically affected acts of violence and minor misdemeanours or petty offenses, reconciliation or mediation may be possible with an agreement from both parties. The family members may choose any option by asking the… Buddhist monk… to act as an arbitrator to solve the problem in order to preserve the harmony within the household in accordance with the nation’s good custom and tradition.’ Thought needs to be given to whether customary modes of reconciliation (*sɑmroh sɑmruəl*) need to be reworked rather than abolished. Monks could do more than smooth the waters; they could catalyse change by promoting Buddhist dhamma with a human rights face. We need to draw inspiration from the seminal work by scholars such as Sally Engle Merry on these matters, when she wrote that ‘Describing some forms of gender violence as “harmful” traditional practices locates them in an unchanging culture implicitly assumed as backward and needing the “civilizing processes” of modernity (Engle Merry 2011:26). Noting the danger that global feminism at times can echo these older missionary ideals, Merry notes that, on the other hand, it can ‘reform and transform gender hierarchies’. In this paper, I argue that this transformation can be achieved within the culture and in such a way that the fabric of the community is not torn further while the rights of women are championed.

## Conclusion

There is a need for interventions to work with tradition and culture rather than only highlight it in problematic terms. Rather than foisting ‘foreign’ ideas on people, change has to harness rather than eradicate the traditional currents of violence that flow in the cultural stream. There is an argument for the value of epigenetic landscapes as an analytical tool.

Understanding the cultural epigenesis of GBV in Cambodia leads to six outcomes. (1) An evidence base for cultural determinants of GBV: The cultural epigenesis maps the spiritual, cultural and psychic idioms of GBV and changes our understanding of it. Local and Buddhist concepts that, to the NGO sector, might seem old-fashioned or opaque are turned into more easily grasped practical messages that can be taken up by all stakeholders. (2) A cultural blueprint for sustainable development: The cultural epigenesis of GBV is a blueprint that opens new possibilities for achieving the UN’s Sustainable Development Goal 5 (Gender Equality) by 2030 in a range of cultural settings and evaluated over time in longitudinal, downstream measures. (3) Culture as the solution rather than the problem: By discovering how culture can be part of the solution, rather than the core of the problem, the cultural epigenesis places culture and GBV in a relationship that not only makes better sense, but opens possibilities for using cultural capital to the best effect in the primary prevention of GBV. This is no small matter in a country such as Cambodia with its potential of an estimated 100,000 monks immersed in the teachings of non-violence. (4) Effective translation: The cultural epigenesis responds to the tacit tension between ‘universal rights-based’ and ‘culturally tuned’ approaches to prevention of GBV. It charts a ‘Middle Way’ to the best of both paradigms and highlights common ground between the cultural and international rights approaches to preventing GBV. Translating Buddhist concepts of violence into effective materials, such as curriculum resources and standards for agencies to mitigate violence without trespassing on ‘codes of good habits’, contributes to prevention of GBV. (5) Enrichment of the national capacity for primary prevention of GBV: The cultural epigenesis adds to the evidence needed for the Technical Working Groups on GBV and in clinical handbooks being developed by agencies such as UNFPA/Ministry of Women’s Affairs and UNICEF/Ministry of Health, focusing respectively on GBV against women and girls. International development agencies advocate that training materials should be tailored to the specific socio-cultural context (Bell and Butcher 2015), but they seldom are, and the cultural epigenesis responds to this call. (6) A culturally responsive Theory of Change: The cultural epigenesis offers a new way of looking at Theory of Change. It changes the relationship of all stakeholders, bringing cultural experts such as monks into all activities and bringing a culturally coherent language to prevention, counselling and law enforcement, enabling police, counsellors, educators, mental health professionals and human rights organisations to act as agents for primary prevention. It is hoped that this Theory of Change will influence national capacity during the next decade to harness local culture for primary prevention.
